# Early T-Cell Precursor Acute Lymphoblastic Leukemia: Diagnosis, Updates in Molecular Pathogenesis, Management, and Novel Therapies

**DOI:** 10.3389/fonc.2021.750789

**Published:** 2021-11-29

**Authors:** Chun-fung Sin, Pui-hei Marcus Man

**Affiliations:** Department of Pathology, University of Hong Kong, Hong Kong, Hong Kong SAR, China

**Keywords:** ETP-ALL, T lymphoblastic leukemia, early T-cell precursor acute lymphoblastic leukemia, diagnosis, molecular pathogenesis, novel therapies

## Abstract

Early T-cell precursor acute lymphoblastic leukemia (ETP-ALL) is a distinct subtype of T lymphoblastic leukemia (T-ALL) identified in 2009, due to its unique immunophenotypic and genomic profile. The outcome of patients was poor in earlier studies, and they were prone to have induction failure, with more frequent relapse/refractory disease. Recent advances had been made in discoveries of genetic aberrations and molecular pathogenesis of ETP-ALL. However, the diagnosis and management of ETP-ALL is still challenging. There are limited choices of novel therapies so far. In this review article, it highlighted the diagnostic issue of ETP-ALL, pitfall in diagnosis, and strategy of accurate diagnosis. The review also summarized current understanding of molecular mechanism of leukemogenesis. The emerging role of risk-adapted therapy and allogenic stem cell transplant in optimizing the outcome of patients with ETP-ALL was discussed. Finally, some potential novel therapies were proposed based on the current understanding of molecular pathogenesis.

## Introduction

T lymphoblastic leukemia (T-ALL) is an aggressive hematological malignancy, which accounts for 15% of pediatric ALL and 25% of adult ALL cases (1). Outcomes are favorable in the pediatric population with 75%–80% achieving long-term survival (2). However, the prognosis is particularly poor in the adult population with only 50%–60% achieving long-term survival (3). Moreover, a substantial proportion of patients relapse after chemotherapy and the outcome following relapse/refractory disease is dismal.

Early T-cell precursor acute lymphoblastic leukemia (ETP-ALL) is a distinct entity as defined by World Health Organization (WHO) classification 2017 version due to its unique immunophenotypic and genomic profile compared with other subtypes of T-ALL (4). This entity comprises 5%–17% and 7.4% of pediatric and adult ALL, respectively (5). Recent discovery of genetic aberrations in ETP-ALL will help to better understand its molecular pathogenesis. However, the diagnosis and management of ETP-ALL is still challenging, especially for relapse/refractory disease. ETP-ALL has been reported to have adverse outcome in earlier studies for pediatric and adult patients compared with other subtypes of T-ALL. This review will summarize the diagnostic features of ETP-ALL, the current understanding of genetic aberrations, and its molecular pathogenesis. Furthermore, the role of risk-adapted therapy and allogenic stem cell transplant in alleviating the adverse outcome of ETP-ALL is discussed. Finally, it will highlight potential novel therapies.

## Diagnosis and Pitfall

### Difficulties in Defining ETP-ALL by Immunophenotype

Coustan-Smith et al. first identified cases of ETP-ALL by comparing the gene expression profile of T-ALL cases with that of earliest thymic precursors (ETPs) in mice. ETP-ALL had a characteristic immunophenotype: CD1a and CD8-negative, CD5-negative, or dim (<75% of blasts positive) with one or more stem cell/myeloid markers positive (≥25% of blasts positive) (6). This served as the rationale for defining ETP-ALL under the latest WHO 2017 classification: positive intracytoplasmic CD3 expression and CD7 expression, CD1a-negative, CD8-negative, negative or dim expression of CD5 (<75% positive), and positive (≥25% of blasts population) for at least one stem cell or myeloid antigen, including CD34, HLA-DR, CD13, CD33, CD117, CD11b, and CD65 (4, 7). CD2 and CD4 may also be positive in ETP-ALL under the latest WHO 2017 classification (4).

**Figure 1:**
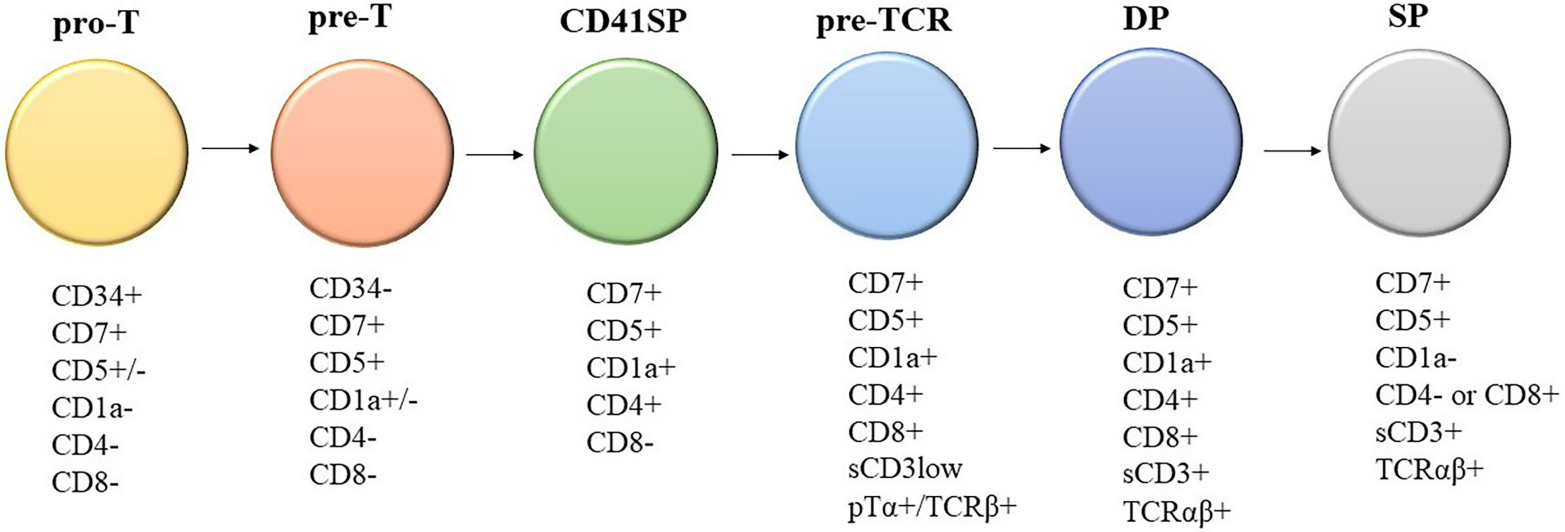
Different stages of T-cell development and their corresponding immunophenotypes: Pro-T, pre-T, immature CD4 single positive (CD4ISP), pre-T-cell receptor (pre-TCR), CD4 and CD8 double positive (DP), and single positive (SP). sCD3: surface CD3; pTα+/TCRβ+: Pre-T-cell receptor alpha and TCR beta chain.

Knowledge of the expression of immunological markers during different stages of T-cell development formed the basis of the European Group on Immunological Classification of Leukemia (EGIL) classification of T-ALL (**Figure 1**) (8). Under the EGIL classification, T-ALL was defined as CD3-positive and TdT-positive and was then further subclassified into T-I (Pro-T-ALL) to T-IV (Mature T-ALL) according to different immunophenotypic profile (**Table 1**) (9, 10). In the EGIL classification, there was no specific entity for ETP-ALL but early thymocytes were classified as CD1a-negative, CD4-negative, and CD8-negative (CD1a-CD4-CD8-) (11). Although the EGIL classification did not present a distinct entity of ETP-ALL, all cases of T-I ALL and some of the T-II ALL cases could also represent ETP-ALL.

**Table 1:** EGIL Classification of T-ALL (8–10).

Subtype	Pattern of immunophenotypic markers expression					
	cCD3	CD7	CD2	CD5	CD1a	Surface CD3
Pro-T-ALL	+	+	-	-	-	-
Pre-T-ALL	+	+	+	+	-	-
Cortical T-ALL	+	+	+	+	+	+/-
Mature T-ALL	+	+	+	+	-	+

A different scoring system was proposed for diagnosing ETP-ALL in the study by the Tokyo Children’s Cancer Study Group. The group devised a scoring system using a 6-marker or 11-marker combination and was validated with a cohort of ETP-ALL patients from Coustan-Smith et al. (**Tables 2**, **3**) (6, 12). The scoring system utilized an extended panel of markers including CD2, surface CD3, CD4, CD10, and CD56 such that it contained an 11-marker combination for scoring. The rationale of the scoring system was that the frequency of CD2, surface CD3, CD4, and CD10 expression was much less frequent from the findings reported by Coustan-Smith et al. (12) Zuurbier et al. evaluated 117 patients with T-ALL and found that the genetic signature of ETP-ALL was best correlated with the following immunophenotype: CD1a-negative, CD4 and CD8-double negative, CD34-positive, and/or CD13/CD33-positive. This immunophenotypic profile had a sensitivity of 77% and a specificity of 94% in identifying ETP-ALL. CD5 status was not considered in this study (13). Khogeer et al. included adult ETP-ALL patients and derived a scoring system utilizing 11 markers. In that scoring system, the expression of surface CD3 (>20% of blasts) and CD5 (>75% of blasts) contributed “−2” marks and a score of ≥8 favored the possibility of an ETP-ALL diagnosis (14).

**Table 2:** Scoring system based on six-marker combination (12).

Marker	-1	+1
CD5	≥5% positive	<75% positive
CD8	≥5% positive	<5% positive
CD13		≥25% positive
CD33		≥25% positive
CD34		≥25% positive
HLA-DR		≥25% positive

Using a score of 4 or more to define ETP-ALL, the sensitivity and specificity were 77% and 100%, respectively.

**Table 3:** Scoring system based on 11-marker combination (12).

Marker	-2	-1	+1	+2
CD5	≥75%			<75% positive
CD8	≥5% positive			<5% positive
CD13			≥25% positive	≥75% positive
CD33			≥25% positive	≥75% positive
CD34			≥25% positive	≥75% positive
HLA-DR			≥25% positive	≥75% positive
CD2		≥75% positive	<20% positive	
CD3		≥75% positive	<20% positive	
CD4		≥75% positive	<20% positive	
CD10		≥75% positive	<20% positive	
CD56			≥20%	

ETP-ALL had a score >6 with a sensitivity of 100% and a specificity of 94%.

The expression of CD5 in ETP-ALL is variable and cases with strong CD5 expression are classified as near-ETP-ALL. One study included 69 pediatric and adult patients with the diagnosis of T-ALL. A definite ETP-ALL phenotype was defined by the WHO criteria. The study showed that ETP-ALL associated with more frequent induction failure, and poorer event-free survival (EFS) and overall survival (OS) compared with other subtypes. However, the study found that 91% of T-ALL cases with immunophenotype CD5+CD1a-CD8- expressed myeloid or stem cell antigens, that is, near-ETP-ALL. The rate of induction failure was significantly higher in this subgroup of T-ALL, despite a non-significant difference of EFS and OS compared with other subtypes. They concluded that cases of near-ETP-ALL behaved like ETP-ALL (15). However, the study did not perform any genomic profiling to verify the ETP-ALL nature of these cases.

A study by Van Vlierberghe et al. in 2011 analyzed 57 adult T-ALL patient samples treated with the Eastern Cooperative Oncology Group (ECOG) E2993 protocol. They found that a number of transcriptionally well-defined early immature T-ALL cases with differentiation block of thymocytes at CD4 and CD8 double-negative stage showed strong CD5 positivity (11% of T-ALL cases) (16). Morita et al. recruited 171 pediatric and adult patients with T-ALL or T lymphoblastic lymphoma (T-LBL). They found that the cases of ETP-ALL and near-ETP-ALL showed certain similarity in their genetic profile, except that myeloid-associated mutations were particularly enriched in ETP-ALL. Moreover, the 5-year OS between near-ETP-ALL and non-ETP-ALL were similar, with significantly worse 5-year OS in the ETP-ALL subgroup (17). Another study showed that ETP-ALL had a distinct genomic profile with enrichment of *LMO2*/*LYL1* aberrations while near-ETP-ALL and non-ETP-ALL enriched with *TAL1* dysregulation (18). These studies showed that both ETP-ALL and near-ETP-ALL share a certain degree of biological similarity and yet may exhibit different genetic profiles and clinical behavior.

The classification of ETP-ALL by immunophenotyping alone is complicated by the different scoring systems available, technical pitfalls in performing flow cytometry, and variability in interpretating flow cytometry results. Moreover, no single diagnostic criterion is perfect in having both high sensitivity and specificity. Recent studies of the genomic landscape of ETP-ALL provide insights into classification, although no specific genetic aberrations can distinguish ETP-ALL from other subtypes of T-ALL to date (18). Noronha et al. found that the *FBXW7* mutation, *CDKN2A/B* deletion, and *STIL*-*TAL1* fusion were only present in ETP-ALL, and these mutations could be used to differentiate ETP-ALL and mixed phenotype acute leukemia with the T/myeloid phenotype (19). Recent studies have proposed a classification of acute myeloid leukemia (AML) and ALL by genetic profile clustering. They showed that more than 15% of leukemia cases could not be reliably classified into either AML or T-ALL and they defined these cases “AML-like T-ALL” and the category was associated with poor prognosis. It is important to note that not all cases of AML-like T-ALL show immature immunophenotype or ETP-ALL phenotype as defined by immunophenotypic criteria (20). Furthermore, single-cell RNA sequencing has demonstrated the expression of signature genes with a spectrum of hematopoietic cells (e.g., mature thymocytes, hemopoietic stem cell, multipotent progenitors, and granulocytic-macrophage progenitors) within the same ETP-ALL cell (21). This suggested that classification of ETP-ALL by conventional methods including flow immunophenotyping may not be robust enough for treatment planning and disease prognostication.

### Technical Considerations for Diagnosing ETP-ALL by Flow Cytometry

Another challenge of diagnosing ETP-ALL is the presence of significant myeloid differentiation in ETP-ALL, and thus, the diagnosis may be confused with acute undifferentiated leukemia or mixed phenotype acute leukemia with T/myeloid phenotype. The availability of a comprehensive panel of monoclonal antibodies and proper interpretation of flow cytometry results are prerequisites for accurate diagnosis.

The Italian Association of Pediatric Hematology and Oncology–Berlin Frankfurt Munich (AIEOP-BFM) recommend that the flow cytometry panel must include antibodies of all hemopoietic cell lineages to exclude the differential diagnosis of acute undifferentiated leukemia or mixed phenotype acute leukemia with T/myeloid phenotype. Thus, a panel of monoclonal antibodies was recommended by AIEOP-BFM for diagnosing pediatric ALL, and the same principles can be extended to the diagnosis of adult ALL (**Table 4**) (4, 22). To confirm the T-lineage nature of the blast population, the combination of cytoplasmic CD3, CD7, and MPO must be used according to AIEOP-BFM recommendation. The T lineage nature of blasts is defined by cytoplasmic CD3 and CD7 positivity while MPO is negative (22). The correct interpretation of cytoplasmic CD3 is the key to a correct diagnosis since it defines the T lineage of blasts. To determine the positivity of cytoplasmic CD3, we should compare staining of a negative internal control sample (e.g., normal B lymphocytes, granulocytes, and monocytes) and positive internal control (normal T cells). At least a fraction of blasts should have positivity for cytoplasmic CD3, with an intensity that is comparable to that of normal T cells, in order to consider that blasts are of T lineage (23). If bone marrow trephine biopsy is available, confirmation of CD3 positivity by immunohistochemical staining on trephine biopsy is preferable (24). A minor proportion of blasts have weak positivity towards cytoplasmic CD3 with an intensity just above the negative control and are not sufficient to conclude the T lineage of blasts, since some cases of AML also have this degree of cytoplasmic CD3 expression. (23) We suggest that diagnostic laboratories should follow the above recommendations for establishing the diagnosis of ETP-ALL from flow cytometry.

**Table 4:** AIEOP-BFM consensus antibody panel for pediatric ALL (22).

Intracellular antigens	CD3, CD22, CD79a, cytoplasmic Mu-chain, MPO, lysozyme (if available)
Surface antigens	CD2, CD3, CD5, CD7, CD10, CD19, CD20; CD11b, CD11c, CD13, CD14, CD15, CD33, CD64, CD65, CD117; CD34, CD56, HLA-DR For T-ALL: CD1a, CD4, CD8, TCR alpha/beta, TCR gamma/delta For B-ALL: Kappa and lambda light chain

Needs to be combined with CD45.

In summary, there are two approaches in the diagnosis of ETP-ALL: (1) classification by gene expression profiling or (2) diagnostic criteria by flow cytometry immunophenotyping. For the routine practice of the diagnostic laboratory setting, the first approach is not feasible; thus, we must rely on flow immunophenotyping for diagnosis despite its limitations. The purpose of flow cytometry is not only to diagnose ETP-ALL, but also to establish the diagnosis of acute leukemia among all differential diagnosis of hematological malignancies as well as lineage assignment. Thus, performing immunophenotyping using the comprehensive antibody panel recommended by the AIEOP-BFM is essential for all laboratories to improve diagnostic accuracy (**Table 4**). The drawback of various scoring systems is that they exclude some of the antibodies recommended by the AIEOP-BFM and the diagnostic sensitivity and specificity are not validated after incorporating the information given by all those antibodies in AIEOP-BFM flow cytometry panel (22). The WHO classification, which is based on the study by Coustain-Smith et al., includes the most extensive panel of markers among all systems of classification discussed above. In addition, the proposed immunophenotypic criteria have been well-validated by gene expression profiling. Thus, we recommend a diagnostic approach (**Table 5**) based on the backbone of the WHO criteria and knowledge of marker expression of various stages of T-cell development (**Figure 1**) (8). The definition of CD1a-, CD8-, CD5 negative or dim, surface CD3, and T-cell receptor expression negative matches the pattern of expression of T cells earlier than the pro-T-cell stage.

**Table 5:** Suggested diagnostic criteria for ETP-ALL.

ALL cases must be positive for cytoplasmic CD3 positive and CD7 plus the following:	
Mandatory criteria (All of the criteria must be present):
	1. CD1a negative
	2. CD8 negative
	3. Surface CD3 negative
	4. TCR alpha/beta and TCR gamma/delta negative
	5. CD5 negative or dim (<75% of blasts positive)
	6. One or more stem cell/myeloid antigens as stated in WHO classification (CD34, HLA-DR, CD13, CD33, CD117, CD11b, and CD65)
Positivity of these markers do not exclude the diagnosis of ETP-ALL	CD2, CD4, CD10

## The Cellular Origin of ETP-ALL and Underlying Molecular Pathogenesis

Previous studies have identified that the earliest thymic progenitors (ETPs) were derived from multipotent bone marrow progenitor cells, rather than common lymphoid progenitors. They retained T and myeloid differentiation potential with B-cell differentiation potential lost either prethymically or intrathymically (25, 26). The origin of ETP-ALL has been suggested to be from ETPs since the gene expression profile of ETP-ALL highly resembled that of ETPs (6). Ugal et al. found that by introducing a *KRAS* mutation and then *KMT2A*-*ENL* rearrangement in bone marrow multipotent granulocyte-monocyte-lymphoid progenitors (GMLPs), mice would develop T-ALL (27), which supports the hypothesis of a cellular origin of ETP-ALL from bone marrow multipotent progenitors. A recent study performed on mouse models introduced oncogenic mutations in early lympho-myeloid progenitors, but not in hematopoietic stem cells (HSCs) or multipotent progenitors, and resulted in the development of T-ALL with the ETP-ALL phenotype (28). The results of these studies demonstrated the cellular origin of ETP-ALL using an *in vivo* model, which was not inferred from the immunophenotypic and genomic profile of ETP-ALL.

Due to the unique cellular origin of ETP-ALL, its genetic profile differs from that of other subtypes of T-ALL in which the transcription profile was similar to that of HSCs of myeloid leukemia (6, 29). Genes encoding transcription factors for development and differentiation, kinase signaling, and epigenetic modifiers are commonly mutated in ETP-ALL. **Table 6** summarizes some of the genetic aberrations found in ETP-ALL. *NOTCH1* mutation, which is a common mutation in other subtypes of T-ALL (31), is less frequent in ETP-ALL and rather more frequent is the presence of *FLT3* and *DMNT3A* mutations (5). Moreover, several recurrent mutations previously not identified in T-ALL were also identified, including *IL7R*, *JAK3*, *RUNX1*, *EZH2*, and *EED (* 29 *).* Mutations in epigenetic regulators (*EZH2*, *PHF6*, and *SUZ12*) were more prevalent in ETP-ALL (32, 33). Moreover, gene rearrangement, e.g., *MEF2C* rearrangement and *KMT2A* rearrangement, is also identified in ETP-ALL (13, 19, 32, 34). The molecular mechanisms of how these mutations lead to leukemogenesis have become clearer with recent pre-clinical and clinical studies.

**Table 6:** Example of genetic aberrations in ETP-ALL (18, 29, 30).

Gene	Type of aberration
**Transcription factors**	
ETV6	Inactivating mutations/deletions
GATA3	Inactivating mutations/deletions
HOXA	Chromosomal rearrangements/inversions/overexpression
LMO2	Chromosomal rearrangement/deletions/overexpression
RUNX1	Inactivating mutations/deletions
WT1	Inactivating mutations/deletions
**Signaling pathway**	
FLT3	Activating mutations/internal-tandem repeat
JAK1	Activating mutations
JAK3	Activating mutations
IL7R	Activating mutations
KRAS	Activating mutations
NRAS	Activating mutations
**Epigenetic modifiers**	
DNMT3A	Inactivating mutations
EED	Inactivating mutations/deletions
EZH2	Inactivating mutations/deletions
PHF6	Inactivating mutations/deletions
SUZ12	Inactivating mutations/deletions
**Genetic rearrangement**	
STIL-TAL1 fusion MEF2C rearrangement KMT2A rearrangement NUP98 rearrangement	

### Mutations in Transcription Factors and Leukemogenesis of ETP-ALL

*LMO2* is a transcription factor belonging to the *LIM*-only class. Expression of *LMO2* in thymocytes increases transcription of HSC-associated genes and repression of genes responsible for T-cell development (35). Besides *LMO2* overexpression, *LYL1* is needed to promote abnormal self-renewal and induce the differentiation block in thymocytes and are frequently co-expressed in ETP-ALL (36). Overexpression of *LMO2* and *LYL1* was present in 36.8% of ETP-ALL in one study (32). *BCL11B* is another transcription factor that is expressed in early T cells and an increase in its expression is associated with irreversible T-cell lineage commitment and differentiation of T cells into CD4 and CD8 double-positive cells (37, 38). Mutation of *BCL11B* causing its under-expression was reported in ETP-ALL (29, 32, 39). *RUNX1* is important in the regulation of thymocyte differentiation and a loss-of-function mutation/deletion of *RUNX1* leads to maturation arrest of thymocytes (40). *GATA3* was shown to be important for the development of ETPs and differentiation to mature T cells (41). Approximately 33% of adult ETP-ALL lack *GATA3* expression, which is mainly due to hypermethylation (42).

### Genetic Rearrangement and Leukemogenesis in ETP-ALL

*MEF2C* rearrangement or rearrangement involving *MEF2C*-related cofactors accounts for approximately half of ETP-ALL cases (43). *MEF2C* rearrangement results in upregulation of *MEF2C* expression, which leads to differentiation block of early thymocytes by upregulating *LMO2* and *LYL1*. Additional genetic aberrations in *RAS* and *MYC* promote the development of the leukemic phenotype (34). *KMT2A* rearrangement results in the upregulation of *HOXA*-related genes in T-ALL (44), which was found to cause differentiation block of progenitor cells and promote the leukemic phenotype (45). Furthermore, the overexpression of *HOXA* in adult T-ALL was shown to be associated with the ETP-ALL phenotype. ETP-ALL with *HOXA* overexpression was also associated with poor survival and increased relapse rate (46). Introducing *KMT2A*-*ELN* fusion in bone marrow progenitor caused differentiation block of thymocytes and the addition of *RAS* mutation promoted leukemic phenotype (27).

### Activating IL7R Mutations and Leukemogenesis in ETP-ALL

*IL7R* mutations are common in adult ETP-ALL (45% of ETP-ALL) and are associated with slow response to chemotherapy. *IL7R* mutations also constitute 8% of pediatric ETP-ALL (29, 47). Moreover, mutations in the *IL7R* pathway were also associated with *PRC2* mutations in T-ALL (48).

**Figure 2:**
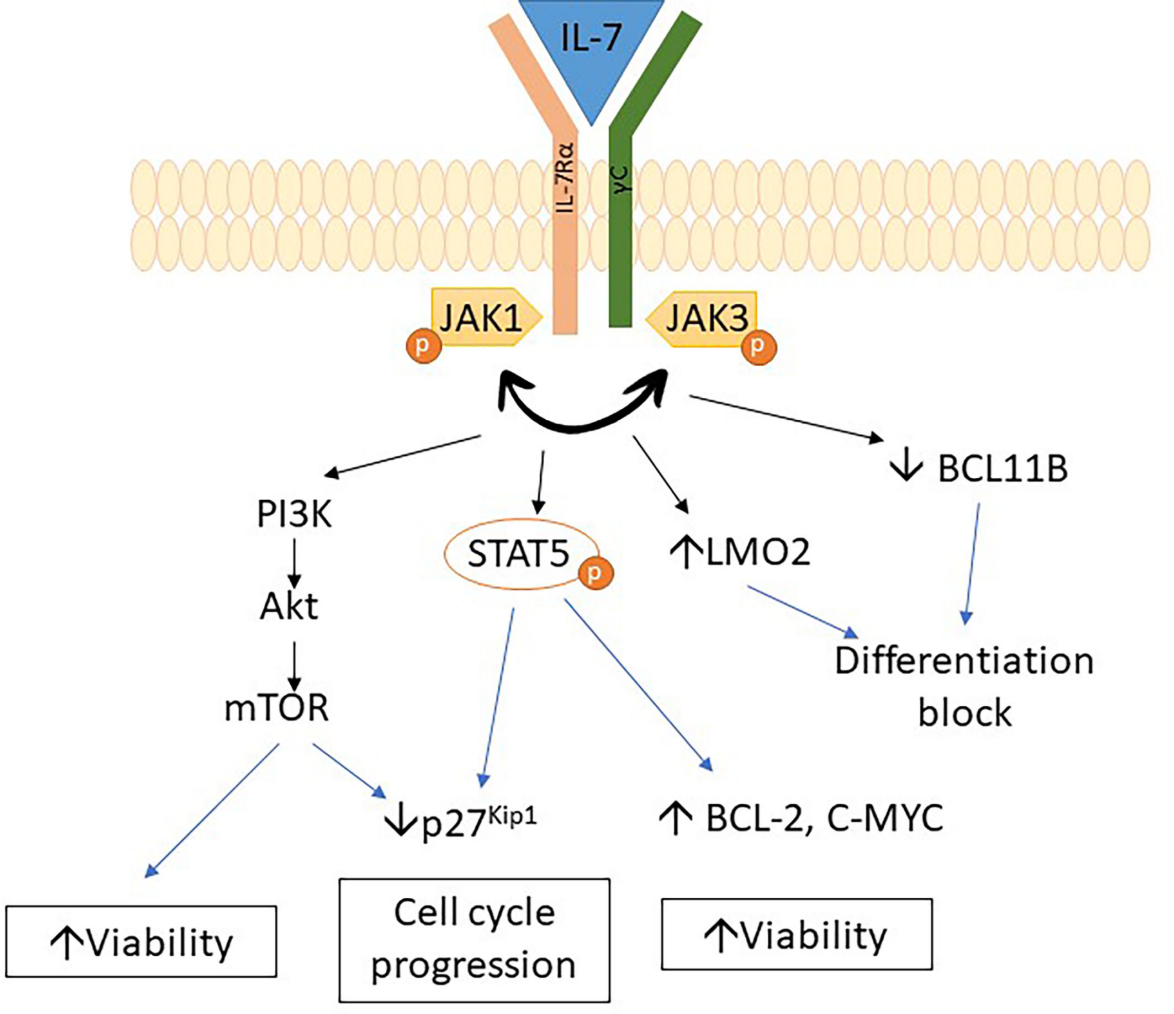
Schematic diagram representing the mechanism of leukemogenesis of *IL7R* mutations.

*IL7R* encodes the IL7 receptor alpha chain, which is important for early lymphoid maturation (49). *IL7R* mutations trigger autonomous homodimerization with activation of the receptor and subsequent phosphorylation of STAT5. It results in dysregulation of various pathways, such as the upregulation of *BCL2* expression, downregulation of cyclin-dependent inhibitor *p27* ^kip1^, and hyperactivation of the PI3K/Akt/mTOR and JAK/STAT pathways, with subsequent progression to leukemic phenotype (29, 47, 50–55). A recent study showed that *IL7R* mutations upregulate *LMO2* expression while downregulating *BCL11B* expression and lead to a differentiation block in thymocytes. Upregulation of myeloid-associated genes was also observed in thymocytes due to *IL7R* mutations (50). The above studies indicate that mechanisms involved in leukemogenesis associated with *IL7R* mutation are due to differentiation block of thymocytes as well as dysregulation of pathway affecting growth and apoptosis (**Figure 2**).

### Mutations in Epigenetic Regulators in Leukemogenesis of ETP-ALL

**Figure 3:**
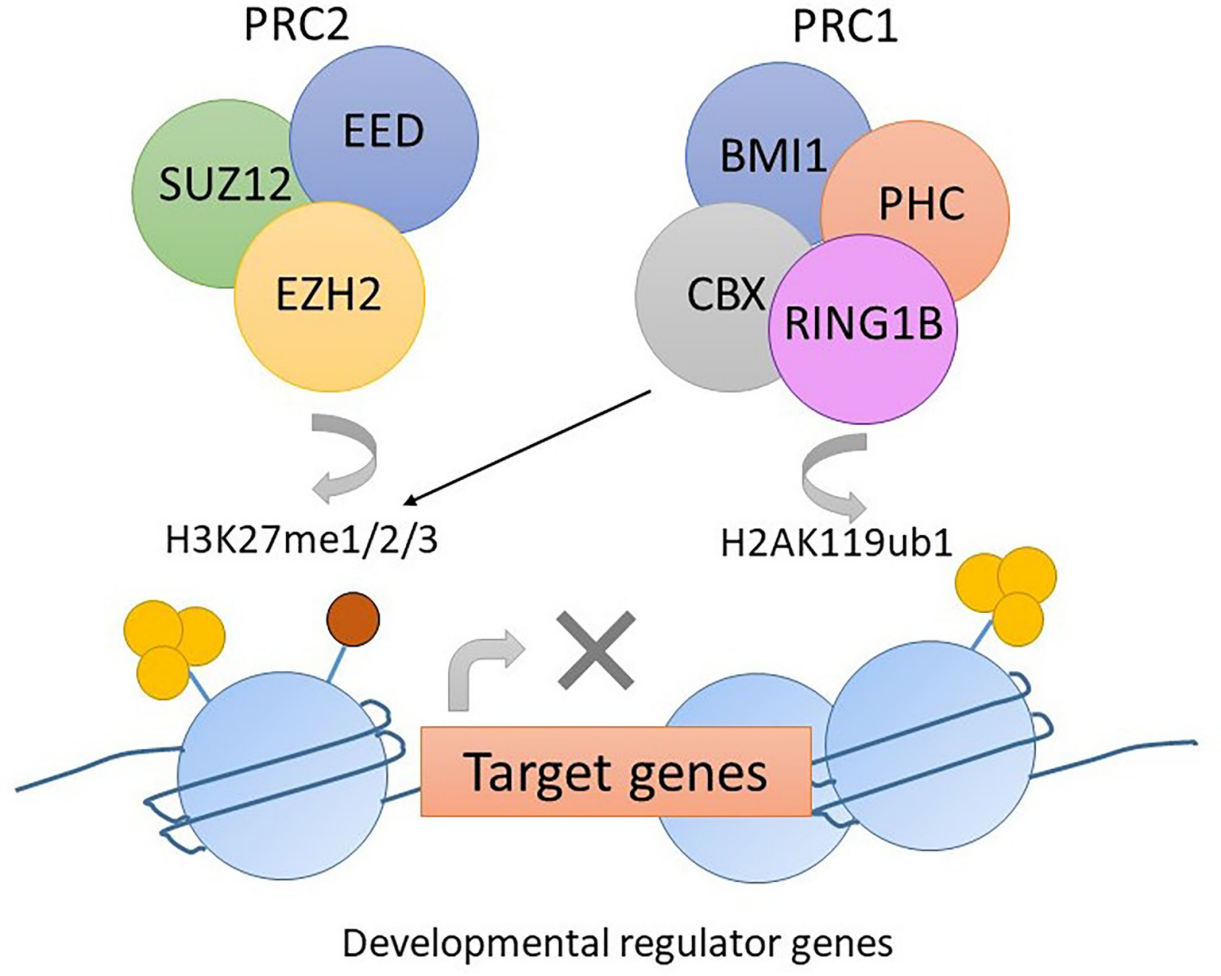
The function of PRC2 complex in normal hemopoiesis. EZH2 mediates the methylation of lysine 27 of histone 3 tail (H3K27me3). The histone modification H3K27me3 recruits PRC1. CBX in PRC1 mediates mono-ubiquitylation of lysine 119 of histone 2A (H2AK119ub). Gene transcription is suppressed due to histone modification.

Polycomb repression complex 2 (PRC2) is a protein complex regulating histone modifications and expression of developmental genes. EZH2 is a component of PRC2 that carries a SET domain involved in methylating lysine 27 of the histone 3 tail (H3K27me3). This histone modification recruits PRC1, which then condenses the chromosome and suppresses gene transcription (**Figure 3**) (56). Mutations in PRC2 components occur in up to 48% of pediatric ETP-ALL with the most common mutation being a loss-of-function mutation in *EZH2*, followed by *SUZ12* and *EED* (29).

**Figure 4:**
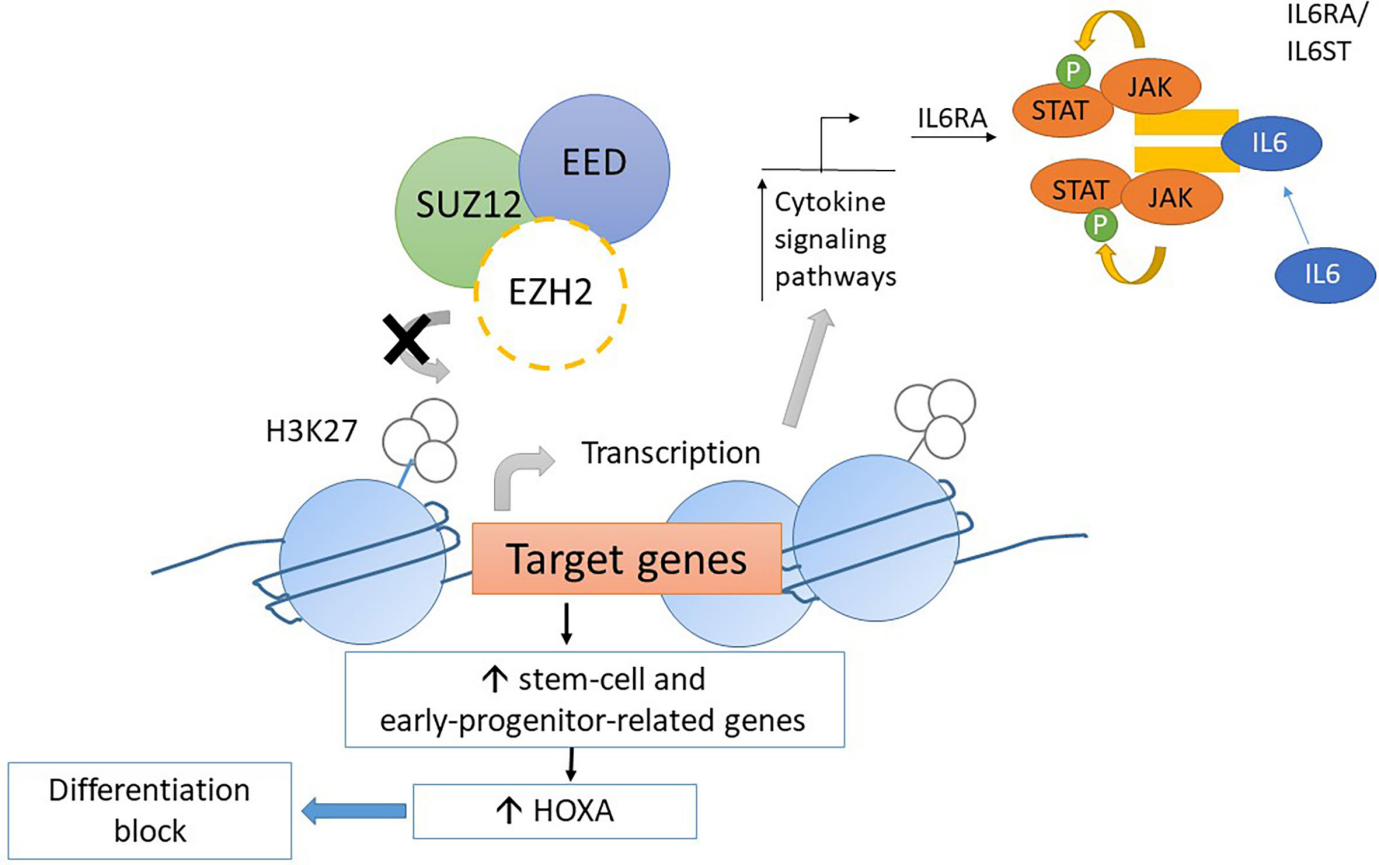
Mechanism of leukemogenesis due to loss-of-function mutation in *EZH2*. Loss-of-function mutations in *EZH2* increase transcription of stem-cell and early-progenitor related genes including *HOXA*. It results into differentiation block of thymocytes. Moreover, the transcription of *IL6RA* is increased due to loss-of-function *EZH2* mutations. Thus, the expression of IL6 receptor is increased and causes hyperresponsiveness to IL6 which results into hyperactivation of STAT3.

Mice transplanted with *CDKN2A* ^-/-^/*NRAS* Q61K/*EZH2* ^Δ/Δ^ mutated lineage-negative, SCA-positive, and c-Kit-positive (LSK) cells generate acute leukemia with the ETP-ALL phenotype. The homozygous loss of *EZH2* resulted in an increase in the transcription of genes relating to stem-cell and early-progenitor-cell programming with a differentiation block at the ETP stage. This results in hyperactivation of the JAK/STAT signaling pathway *via IL6RA* and *STAT3* upregulation (**Figure 4**). *HOXA* is upregulated as well (57). Similar findings resulting from *EZH2* knockout were found by implanting *EZH2* ^Δ/Δ^ *RUNX1* ^Δ/Δ^ mutated lymphoid progenitor cells in mice in another study. However, the *FLT3*-ITD mutation was needed for progression to acute leukemia with ETP-ALL phenotype. The addition of *FLT3*-ITD mutation was also associated with upregulation of genes for *RAS* signaling and *IL7R* (28). Loss-of-function mutations in *EZH2* also associate with DNA hypermethylation. The transcription of various genes, for example, *RUNX1*, was reduced due to DNA hypermethylation (58). Further studies are required to elucidate the role of DNA hypermethylation in leukemogenesis.

**Figure 5:**
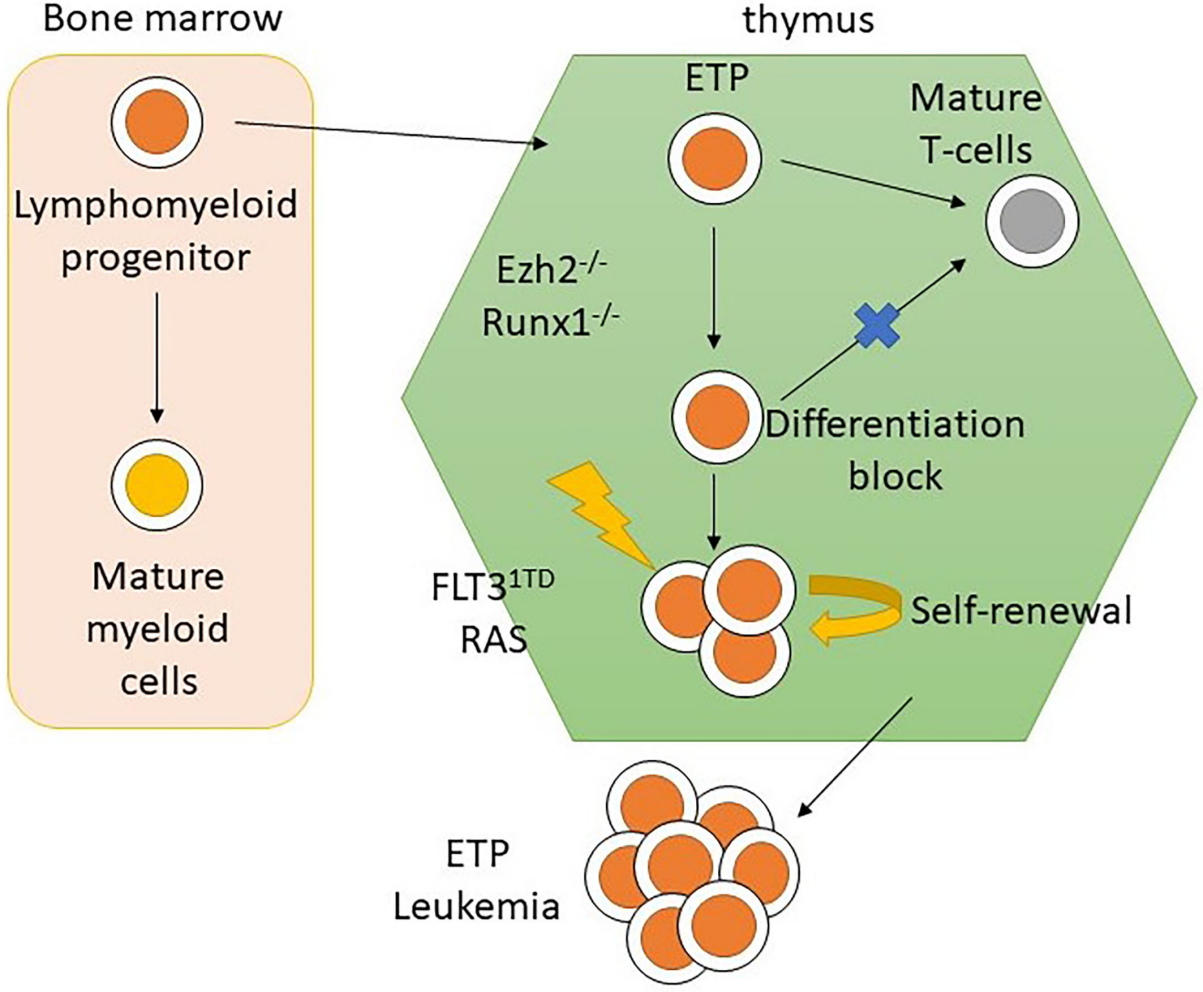
The role of mutation in *EZH2*, *RUNX1*, *RAS*, and *FLT3*-ITD in leukemogenesis of ETP-ALL.

In summary, a loss-of-function mutation of *EZH2* in ETP leads to maturation arrest at the ETP stage by modulating gene expression *via* the reduction of histone methylation and DNA hypermethylation. However, progression to acute leukemia with the ETP-ALL phenotype requires acquisition of other driver mutations, such as *FLT3*-ITD and *NRAS*, that lead to dysregulation of pathways regulating cellular proliferation and apoptosis (**Figure 5**) (28, 57, 58). This provides mechanistic basis for designing targeted therapy to reverse differentiation block and inhibit driver mutations as a possible synergistic treatment regimen.

## Clinical Management of ETP-ALL

Conventional intensive chemotherapy remains the mainstay of treatment for ETP-ALL and poor prognosis was reported by Coustan-Smith et al. compared with non-ETP-ALL (6). However, the prognostic impact of ETP-ALL phenotype alone is controversial from recent studies. **Table 7** summarizes the findings of these clinical studies.

**Table 7:** Clinical studies of evaluating the outcome of ETP-ALL.

Study	Age of patients	Number of patients with ETP-ALL	Study details	Nature of study	Result	Remarks
Coustan-Smith et al. (6)	0.5–18 years	239 with T-ALL, 30 had ETP-ALL signature	Compared outcome between ETP-ALL and non-ALL treated with standard chemotherapy	Retrospective	10-year OS 19% *vs*. 85%, 10-year EFS 22% *vs*. 69% (*p* < 0.0001), for ETP-ALL *vs*. non-ETP-ALL	Nil
Inukai et al. (12)	1–18 years	5 ETP-ALL patients	Compared outcome between ETP-ALL and non-ALL treated with pediatric protocol	Retrospective	1. 4-year EFS 40% (ETP-ALL) *vs*. 70% (non-ETP-ALL), *p* = 0.0142. Overall survival not statistically different	Limited number of patients with ETP-ALL
Allen et al. (59)	1–81 years	7 ETP-ALL patients	Compared outcome of ETP-ALL and non-ETP-ALL treated with conventional chemotherapy	Retrospective	1. Significantly higher relapse rates in pediatric ETP-ALL (HR = 11.63, *p* = 0.025)2. No difference in event-free and overall survival in all age group	1. Limited sample size2. Heterogeneity of treatment regimen
Jain et al. (5)	13–79 years	15 ETP-ALL and 4 T-LBL with ETP-ALL phenotype	Compared outcome of ETP-ALL and non-ETP-ALL treated with augmented BFM or hyper-CVAD	Retrospective	Median OS 20 months *vs*. not reached (*p* = 0.008)	Allogenic stem cell transplant not routinely done for patients with CR1.
Patrick et al. (60)	1–24 years	35 patients with ETP-ALL	Compare outcome of ETP-ALL and non-ETP-ALL treated with UKALL 2003 protocol	Review of data from randomized control trial (UKALL 2003 trial)	Apparently inferior 5-year EFS and OS for ETP-ALL (76.7% *vs*. 84.6%, 82.4% *vs*. 90.9%) but not statistically significant	Adverse prognostic features of ETP-ALL might overcome by risk-adapted therapy with treatment intensification
Sayed et al. (61)	1–18 years	103 patients with T-ALL, 16.5% of them were ETP-ALL	Compared outcome of ETP-ALL *vs*. non-ETP-ALL treated with different protocol	Retrospective	Patients treated with the Total Therapy Study XIII protocol had a non-statistically significant inferior outcome for ETP: 70.8% *vs*. 76.6% (*p* = 0.67)	Adverse prognosis of ETP-ALL might be overcome by risk-adapted treatment intensification
Bond et al. (62)	Adult patients, median age 38.5 years	47 ETP-ALL patients	Compared outcome of ETP-ALL *vs*. non-ETP-ALL treated with pediatric-inspired protocol, an early response-base intensification protocol	Analysis of data from GRAALL – 2003 study (prospective, phase II trial) and GRAALL – 2005 (prospective, randomized control trial)	1. Non-statistically significantly inferior 5-year EFS and OS (59.6% *vs*. 66.5%) in ETP-ALL2. ETP-ALL had inferior 5-year OS without allogenic stem cell transplant (49.2% *vs*. 67.5%, *p* = 0.02)	1. Early response-based intensification could improve outcome of ETP-ALL2. Allogenic stem cell transplant in CR1 could improve outcome of ETP-ALL
Brammer et al. (63)	2–72 years	16 patients with ETP-ALL	Analysis of outcome of different T-ALL subtypes and MRD status after allogenic stem cell transplant	Retrospective	Not statistically different in 3-year OS after allogenic SCT in CR1 (47% *vs*. 65%, *p* = 0.5)	1. Allogenic SCT might overcome adverse prognosis of ETP-ALL2. Limited sample size
S. Fuhrmann et al (64)	1–18 years	493 T-ALL patients in total, 33 patients with ETP-ALL	Analysis of outcome of CD56 expression status in T-ALL from ALL-BFM 2000 trial	Analysis of data from prospective trial (AIEOP-BFM ALL 2000 study)	1. Not statistically significantly different in event-free survival2. CD56 expression had inferior 5-year event-free survival (60% *vs*. 82%, *p* = 0.002%) and 5-year overall survival (60% *vs*. 85%, *p* = 0.003%)	1. 30% of ETP-ALL express CD56 *vs*. 5.1% in non-ETP-ALL2. MRD-directed therapy improved outcome of ETP-ALL
Dunsmore et al (65)	1–31 years	1,596 patients with T-ALL,	Phase 3 RCT of nelarabine randomization in addition to escalating-dose methotrexate (MTX) plus pegaspargase (C-MTX) or high dose methotrexate	Randomized control trial	1. No statistically significant impact on DFS (hazard ratio, 0.99; 95% CI, 0.59 to 1.67; P 5.981; p = 0.981).2. No significant difference in 5-year event-free survival (87% *vs*. 86.9%) and overall survival (93% *vs*. 92%) between ETP-ALL *vs*. non-ETP-ALL.	92 patients with ETP-ALL taken off from the study protocol. Among them, 28 received allogenic hemopoietic stem cell transplant. This might contribute to improve outcome of ETP-ALL
Burns et al. (66)	1–21 years	123 T-ALL patients, 21 patients had ETP-ALL	Analysis of patients enrolled into DFCI 05-001 and DFCI 11-001 trial and identify prognostic factors of T-ALL	Analysis of data from phase III randomized controlled trials (DFCI 05-001 and DFCI 11-001 trial)	1. ETP-ALL associated with higher rate of induction failure (33% *vs*. 5%, *p* = 0.008)2. Inferior 5-year event-free survival for ETP-All (54% *vs*. 87%, *p* = 0.006)3. Not statistically different in 5-year overall survival (85% *vs*. 92%, *p* = 0.31).	1. Adverse prognosis of ETP-ALL could be overcome by treating with high-risk ALL regimen.
Morita et al. (17)	13–78 years, median age 30 years	171 T-ALL patients in total, 21 of them were ETP-ALL	Analysis of outcome of newly diagnosed near-ETP-ALL upon treatment of frontline chemotherapy with or without allo-SCT and effect of nelarabine	Retrospective	1. CR rate of ETP was similar *vs*. near-ETP-ALL and non-ETP-ALL (83 *vs*. 79% *vs*. 91%)2. Inferior 5-year event-free survival of ETP-ALL *vs*. non-ETP-ALL (24% *vs*. 60%, *p* < 0.001) and 5-year overall survival (32% *vs*. 63%, *p* < 0.001)3. Allo-SCT improved 5-year EFS (36% *vs*. 18%, *p* = 0.03).	1 .Allo-HSCT had a trend of better 5-year overall survival in ETP-ALL though not statistically significant (36% *vs*. 29%, *p* = 0.218)2. Allo-HSCT might improve outcome of ETP-ALL
Genesca et al. (67)	Adult T-ALL, mean age 33.5 years	185 T-ALL patients in total, 34 of them were ETP-ALL	Analysis of clinic-biological, outcome, and prognostic features of PETHEMA and ALL-HR-2003 trials	Retrospective	1. ETP-ALL significantly lower CR rate upon induction (77% *vs*. 94%, *p* = 0.005)2. ETP-ALL had lower MRD negative rate after day 35 induction (35% *vs*. 82%, *p* < 0.001, MRD < 0.1% as cutoff)3. Inferior overall survival at 4-year for ETP-ALL (36% *vs*. 49%, *p* = 0.037)	1 .Higher rate of induction failure and slow MRD clearance for ETP-ALL2. The overall survival for patients with allo-SCT was lower in PETHEMA
Conter et al. (68)	1–18 years	139 treated with AIEOP R2006 study, 16 of them ETP-ALL. 201 T-ALL patients from AIEOP-BFM 2009 study, 33 of them were ETP-ALL	Analysis outcome of ETP-ALL patients treated with AIEOP-BFM protocol	Retrospective	1. High rate of MRD positivity (cutoff 5 × 10^−4^): 85% at day 33 and day 78A2 .AIEOP-ALL R2006 study: 5-year event-free and overall survival (56.3% and 55.6%, respectively)3. AIEOP-BFM ALL 2009 study: 3-year event-free and overall survival (86.2%)	1. Slow marrow response and MRD response for ETP-ALL2. Outcome of AIEOP-BFM ALL 2009 study not statistically different from non-ETP-ALL3 .More patients assigned to high-risk treatment protocol in AIEOP-BFM ALL 2009 study due to positive MRD4 .Improve outcome of ETP-ALL after treatment intensification
P. Quist-Paulsen et al. (69)	1–45 years	A total of 278 T-ALL patients, 37 of them were ETP-ALL	Analysis of results of both pediatric and adult patients with ALL treated with NOPHO ALL 2008, a pediatric-inspired protocol	Prospective study of patients treated with NOPHO ALL 2008 protocol	1 .Higher MRD at day 29 (0.3% *vs*. 0.04%, *p* = 0.001)2. Not statistically significantly different in relapse risk and overall survival	Most patients assigned to high-risk protocol or allo-SCT due to poor early MRD response

EFS, Event-free survival; OS, Overall survival; HR, Hazard ratio; CR, Complete remission; RCT, Randomized-controlled trial; allo-HSCT, Allogenic hemopoietic stem cell transplant; MRD, Minimal residual disease.

### Results of Studies Involving Pediatric Patients

A total of 47 pediatric T-ALL patients from the Children’s Oncology Group (COG) 09404 and the Dana–Farber Cancer Institute (DFCI) 00-01 study were included in a study, which showed that the absence of the biallelic T-cell receptor (TCR) gamma deletion was strongly associated with induction failure. This subgroup of T-ALL conferred significantly worse EFS and OS than the other subgroup of T-ALL. Among the 40 patients showing biallelic TCR gamma deletion with gene expression data available, 14 presented features consistent with ETP-ALL. The ETP-ALL phenotype showed similar effects on EFS and OS as biallelic TCR gamma deletion. However, only one patient from the above cohort exhibited an immunophenotype consistent with ETP-ALL, which highlighted that flow immunophenotyping had presented limitations in diagnosing ETP-ALL as mentioned above (70).

The Tokyo Children’s Cancer Study Group (TCCSG) study L99-15 included 91 Japanese patients aged 1–18 years with a diagnosis of T-ALL. Among these, five patients met the diagnostic criteria of ETP-ALL. They were treated with a pediatric protocol described by Manabe et al. (71) The patients were stratified into three risk groups according to blast count in the peripheral blood after 7 days of prednisolone therapy and allogenic hemopoietic stem cell transplant was offered to those in the high-risk group. In the cohort of patients with ETP-ALL, the EFS was significantly worse than in other subtypes (40% *vs*. 70% over 4 years). There was no statistically significant difference in OS between the ETP-ALL and other subtypes. Allogenic HSC transplant might also play a role in alleviating the adverse outcomes of ETP-ALL patients from that study (12). However, the main weakness of the study was the limited number of ETP-ALL patients recruited.

Sayed et al. recruited 103 pediatric patients (aged 1–18 years) with T-ALL from various Arab centers. Among them, 16.5% exhibited the ETP-ALL phenotype and most were treated with St Jude Total Therapy Study XIII for high-risk ALL protocol. There was a trend for a higher rate of induction failure in ETP-ALL versus non-ETP-ALL patients (10% *vs*. 7%, *p* = 0.679). The OS and disease-free survival (DFS) were not statistically different between ETP-ALL and non-ETP-ALL. The study indicated that the adverse outcome of ETP-ALL could be overcome by risk-adapted treatment intensification. However, the power of this study was limited by the small sample size of ETP-ALL patients (61).

A retrospective study included 49 patients treated with the AIEOP-BFM protocol aged 1–18 years old with a diagnosis of ETP-ALL. The AIEOP-BFM protocol is composed of three blocks of polychemotherapy after phase 1B intensification in ALL high-risk group patients, and as defined by poor day 7 prednisolone response, complete remission was not achieved after phase 1A or MRD level ≥5 × 10^–^⁴ after day 78 of treatment. Despite the higher number of patients with blasts ≥10% at day 15 of treatment, poor prednisolone response, complete remission not achieved after phase 1A of therapy, as well as a significant proportion of patients with a MRD level above 5 × 10^–^⁴ after day 33 (85% of patients) and day 78 (20% of patients) of treatment, the 3-year EFS rate was 86%, and it was not significantly different from other subtypes of T-ALL. (68) Thus, the outcome of ETP-ALL improved with treatment intensification in that study.

Pediatric patients from the AIEOP-BFM ALL 2000 trial diagnosed with T-ALL were treated with multi-block chemotherapy and MRD-directed regimens. The study showed that the ETP-ALL phenotype did not affect the EFS and OS significantly. However, the study did find a negative prognostic impact in terms of inferior EFS and OS for CD56-positive T-ALL. Approximately 30% of ETP-ALL patients had CD56 positivity, while only 5.1% of non-ETP-ALL expressed CD56 (64). Similarly, specific genetic markers, e.g., *NOTCH1* mutation, were associated with the patient’s outcome. Noronha et al. found that the presence of *NOTCH1* mutation conferred a favorable prognosis in ETP-ALL with significantly better OS for pediatric patients (19).

A study including a large cohort of 464 pediatric patients with T-ALL showed that ETP-ALL was strongly associated with positive MRD after day 33 and day 78 of treatment, in patients belonging to the MRD high-risk group, and this group also presented a higher incidence of relapse (7-year cumulative incidence of relapse: 44.7%). Allogenic HSC transplant reduced the risk of relapse in these patients compared with chemotherapy alone (72).

### Studies Involving Both Pediatric and Adult Patients

A study by Jain et al. included 111 patients from the MD Anderson Cancer Center with a diagnosis of T-ALL/lymphoblastic lymphoma. Of these, 15 patients fulfilled the criteria of ETP-ALL and 4 patients fulfilled the criteria of T lymphoblastic lymphoma with the ETP-ALL phenotype. In that study, patients were treated with either the hyper-CVAD +/- nelarabine or the augmented Berlin-Frankfurt-Münster (BFM) regimen. The study showed that the ETP-ALL phenotype was associated with inferior outcome than other subtypes of T-ALL. The median OS was 20 months in ETP-ALL versus non-reached in other T-ALL subtypes. Although the status of MRD negativity did not affect the outcome of ETP-ALL, the number of patients with MRD data available was relatively small (5). Morita et al. analyzed data from 171 T-ALL patients from the MD Anderson Cancer Center during the period of 2000–2019, which also included a cohort of patients in the study by Jain et al. (17). Of these, 27 patients had ETP-ALL. Similarly, the study found that ETP-ALL was associated with significantly poor EFS and OS compared with other subtypes of T-ALL. Allogeneic HSC transplant at first remission improved the 5-year EFS (36% *vs*. 18%) significantly with a trend of better 5-year OS (36% *vs*. 29%), although it was not statistically significant. The study revealed the survival benefit of allogenic HSC transplant in ETP-ALL (17).

A retrospective review included 185 adult patients diagnosed with T-ALL from ALL-HR2003 and ALL-HR-11 trials. In that study, 34 patients matched the diagnostic criteria of ETP-ALL. The study showed that the response to induction chemotherapy was significantly poorer than other subtypes of T-ALL. The rate of MRD >0.1% at day 35 of treatment was significantly higher. The ETP-ALL was independently associated with poorer EFS and OS compared with other subtypes of T-ALL (67).

A retrospective study included 48 patients with T-ALL/T lymphoblastic lymphoma in a center in Columbia aged 8 months to 81 years. Among these, 7 patients presented an ETP-ALL phenotype. They were treated with various conventional combination chemotherapy protocols for high-risk ALL. The study showed that the risk of relapse of the ETP-ALL subgroup was significantly higher with inferior EFS in the pediatric population (59). The main limitation of the study was the small patient number as well as the heterogeneity of treatment regimens.

The COG-AALL0434 study enrolled a total of 1,596 patients aged 1–31 years old with the diagnosis of T-ALL, including patients with ETP-ALL. Patients received a prednisolone-based, four-drug combination chemotherapy as the induction regimen. Patients were then stratified into low-risk, intermediate-risk, and high-risk subgroups according to the NCI risk classification, prednisolone response, any central nervous system/testis involvement, and bone marrow response after induction therapy. The intermediate-risk and high-risk groups were randomized for nelarabine therapy and the latter achieved a 5-year OS of 89.5% and the 5-year EFS rate was 83.7%. In total, 92 patients with ETP-ALL were taken off the study protocol. Among these patients, 28 received allogenic HSC transplant. From the multivariate analysis of the overall cohort, ETP-ALL status did not have a significant impact on DFS (65). Again, the study showed the benefits of risk-adapted therapy and allogenic HSC transplant in ETP-ALL.

The phase III clinical trials DFCI 05-001 and DFCI 11-001 recruited a total of 123 patients aged 1–21 years with T-ALL, including 21 patients with the ETP-ALL phenotype. They were treated with prednisolone induction and then multiagent chemotherapeutic drugs. All patients with T-ALL were treated with high-risk arms therapy. A significantly higher rate of induction failure was observed in ETP-ALL patients and thus a significantly poorer 5-year EFS. However, the 5-year OS was not significantly different from non-ETP-ALL. The study showed that the adverse prognostic outcome of ETP-ALL could be alleviated by treatment with a high-risk regimen (66).

The UKALL 2003 study recruited patients aged 1–25 years and 388 T-ALL patients were included. Among these, 35 patients had a diagnosis of ETP-ALL. The trial classified patients into standard-, intermediate-, and high-risk based on clinical factors, early morphological response, and cytogenetics. Those standard-risk patients received a three-drug combination and intermediate- and high-risk patients received a four-drug combination for induction. Patients with standard and intermediate risk were stratified into MRD low-risk and MRD high-risk groups, depending on the MRD level. They were then randomized for treatment reduction (for those with undetectable MRD or MRD <0.01% post-induction but undetectable after consolidation) or who received treatment intensification (MRD >0.01% post-induction) (73). During the median follow-up period of 4 years and 10 months, the 5-year EFS and OS for the ETP-ALL group were not significantly different from other subtypes of T-ALL. The authors attributed the findings to the use of pegylated asparaginase and dexamethasone. Furthermore, the more intensive regimen was given to patients with slow morphological response and the MRD response that occurred more frequently in the ETP-ALL subgroup (60).

A study of 47 adult patients with ETP-ALL was conducted, which included patients from the Group for Research on Adult Acute Lymphoblastic Leukemia (GRAALL)-2003 and -2005 studies. The patients were treated with the GRAALL protocol, a pediatric-inspired protocol. The essence of this protocol is treatment intensification if there is evidence of early treatment resistance, e.g., MRD positivity after induction chemotherapy. In this study, 71.4% of patients with ETP-ALL had MRD level ≥10^-4^ at day 42 after treatment and 50% of patients with MRD level higher than that level after day 84 of treatment were eligible for allogeneic HSC transplant. The 5-year OS was not statistically different from other T-ALL subtypes. Of note, approximately 48.6% of ETP-ALL patients received allogenic stem cell transplant and the ETP-ALL phenotype was associated with better outcome upon allogenic stem cell transplantation. Patients with ETP-ALL achieved an inferior 5-year OS without allogenic stem cell transplant (62).

Data from patients treated with the NOPHO 2008 protocol, an unmodified pediatric protocol that included patients aged 1–45 years old and a total of 278 T-ALL patients, were included. Among them, 37 patients belonged to the ETP-ALL subgroup. Patients were stratified into intermediate- and high-risk groups according to MRD response at day 29. Patients would be eligible for allogenic HSC transplant if MRD was ≥0.1% at day 79 or MRD was >5% at day 29. The results showed that ETP-ALL patients did not achieve a statistically significantly different relapse rate or OS compared with the non-ETP-ALL patients, although they had slow MRD response and thus most patients received high-risk arm therapy or allogenic HSC transplant (69). Again, this study showed an improved outcome for ETP-ALL when treated with risk-adapted therapy and allogenic HSC transplant might have a role in improving outcome of ETP-ALL patients.

Another study involving 16 patients with ETP-ALL who underwent allogeneic stem cell transplant followed by treatment with hyper-CVAD plus or minus nelarabine achieved a 3-year OS of 47% for ETP-ALL patients, which was not statistically different from other T-ALL subtypes (63). However, adult patients with T-ALL carrying *IL7R* mutations did not show improvement in OS after allogenic HSC transplant, in contrast with those patients without *IL7R* mutations. Patients diagnosed as ETP-ALL had a higher rate of *IL7R* mutations in that study, although some patients with non-ETP-ALL phenotype also carried *IL7R* mutations (47).

In summary, evidence from recent studies indicates that risk-adapted therapy with treatment intensification carries survival benefits to both pediatric and adult ETP-ALL patients. Allogenic hemopoietic stem cell transplant may have a role in alleviating adverse outcome of ETP-ALL as is evident from recent studies, especially those involving adult patients. Further studies are needed to evaluate the role of allogeneic stem transplant in managing ETP-ALL, especially in the pediatric population and further research in evaluating the role of different genetic aberrations benefitting from HSC transplant are needed. Some genetic aberrations in ETP-ALL, for example, *NOTCH1* and *IL7R*, carry prognostic impact, and the approach of disease prognostication by various genetic aberrations requires extensive validation in subsequent studies.

## Novel Therapies

Given the dismal prognosis of relapse/refractory disease of ETP-ALL and the challenge in managing patients who cannot tolerate intensive chemotherapy, there is an urgent need to develop novel treatments for ETP-ALL based on the understanding of disease biology and molecular mechanisms. **Table 8** summarizes the novel therapies available for ETP-ALL. **Table 9** summarizes various ongoing clinical trials involving novel therapies.

**Table 8:** Summary of potential novel therapies available for ETP-ALL.

Type of Novel Therapy	Rationale	Therapeutic Target	Single Treatment or Proposed Combined Treatment	Data from Pre-clinical Studies for ETP-ALL Available?	Data from Clinical Studies for ETP-ALL Patients Available (Except Case Reports)?
JAK inhibitor (ruxolitinib)	JAK/STAT pathway hyperactivation is common in ETP-ALL	JAK	Single treatment	Yes	No
Anti-CD33 (gemtuzumab)	CD33 expression is frequently present in ETP-ALL	CD33	Single treatment	Yes	No
Anti-CD38 (daratumumab)	CD38 expression is frequently present in ETP-ALL	CD38	1. Single treatment 2. Combined with nelarabine	Yes	No
Anti-CD123	CD123 expression is prevalent in ETP-ALL	CD123	Single treatment	No	No
CAR-T	Genetically engineered patient’s T cells to target against various antigens present on ETP-ALL	CD5, CD7	Single treatment	Yes	No
Hypomethylating agents (decitabine, azacytidine)	1. DNA hypermethylation associated with PRC2 mutations2. High rate of DNMT2A mutation in adult ETP-ALL	1. Targeting epigenetic regulation of gene transcriptions2. Upregulation of NOXA in AML	Combination therapy with venetoclax or combined chemotherapy	No	No
BCL-2 inhibitor (venetoclax)	ETP-ALL is highly dependent on BCL-2 activity	BCL-2	1. Single treatment 2. Combination therapy with conventional chemotherapy, hypomethylating agents, bortezomib, navitoclax	Yes	Yes
FLT3 inhibitors	FLT3-ITD and FLT3-TKD mutations are common in ETP-ALL	FLT3	Single treatment	No	No
BET inhibitors	Frequent PRC2 mutations in ETP-ALL	BET protein	Single treatment	Yes	No

**Table 9:** Ongoing clinical trials on novel therapies.

Trial	ALL subtype	Age	Phase	Overview
NCT03808610	Relapse/refractory T and B-ALL	≥18 years	Phase I/II	Combination of low-intensity chemotherapy and venetoclax in patients with relapsed/refractory ALL
NCT03504644	Relapse/refractory T and B-ALL	≥18 years	Phase IB/II	Combination of venetoclax and liposomal vincristine in Patients with Relapsed or Refractory ALL
NCT03808610	Relapse/refractory T and B-ALL	≥18 years	Phase I/II	Combination of low-intensity chemotherapy and venetoclax (ABT-199) in patients with Relapsed/Refractory Acute Lymphoblastic Leukemia (ALL)
NCT04752163	Relapse/refractory AML, ALL, CMML and MDS	≥18 years	Open-Label Phase 1/2	Single treatment of DS-1594b or combination with azacytidine and venetoclax or Mini-HCVD for the Treatment of AML and ALL
NCT03236857	Various relapse/refractory malignancies, e.g., AML, ALL, neuroblastoma	Pediatric and young adults up to 25 years	Phase 1	Study of safety and pharmacokinetics of venetoclax in pediatric and young adult patients with relapsed or refractory malignancies
NCT03117751	Newly diagnosed T and B-ALL	1–18 years	RCT	Add ruxolitinib for JAK-STAT hyperactivated ALL in combination with conventional chemotherapy
NCT03384654	Relapse/refractory T and B-ALL	1–30 years	Open-label, phase 2	Investigation of efficacy and safety of daratumumab in pediatric and young adult with relapsed/refractory ALL
NCT03860844	Relapse/refractory AML, T and B-ALL	28 days to 17 years	Open-label, single-arm trial	Evaluation of antitumor activity, safety, and pharmacokinetics of isatuximab used in combination with chemotherapy in pediatric patients with relapsed/refractory ALL or AML
NCT03386513	CD123-positive relapse/refractory hematological malignancies, including AML and ALL	≥18 years	Phase I/II	Determine the maximum tolerated dose, evaluate safety, tolerability, pharmacokinetics, immunogenicity, and anti-leukemia activity of IMGN632 when administered as monotherapy to patients with CD123+ disease.
NCT04681105	CD123-positive relapse/refractory hematological malignancies, including ALL and blastic plasmacytoid dendritic cell neoplasm	≥12 years	Phase 1	Determine the best dose and assess side effect of flotetuzumab in treating relapse/refractory CD123-positive malignancies
NCT03081910	Relapse CD5-positive T-ALL and mature T-cell lymphoma	Pediatric to adults up to 75 years	Phase 1	Phase 1 therapy with manufactured CAR-T cell for treatment of T-cell malignancies expressing CD5 antigen
NCT03690011	Relapse CD7-positive T-ALL and mature T-cell lymphoma	Pediatric to adult up to 75 years	Phase 1	Phase 1 Therapy with manufactured CAR-T cell for treatment of T-cell malignancies expressing CD7 antigen
NCT04004637	Relapsed CD7-positive T-ALL/T lymphoblastic lymphoma, NK/T-cell lymphoma (T-LBL)	7–70 years	Phase 1	Investigate the safety and efficacy of CD7 CAR-T cells for patients with relapse/refractory CD7+ T-ALL/T-LBL, NK/T cell lymphoma and determine the pharmacokinetics of CD7 CAR-T cells in patients.
NCT04860817	Relapse/refractory T-ALL/T-LBL	2–25 years	Early Phase 1	Investigate the safety and efficacy of CD7 CAR-T cell in treating relapse/refractory T-ALL/T-LBL
NCT04620655	Relapse/refractory T-ALL/T-LBL	3–70 years	Early phase 1	Determine the dose-limiting toxicity of universal CD7 CAR-T cell in treating relapse/refractory T-ALL/T-LBL

### Venetoclax

*BCL2* was found to be highly expressed in the DN1 stage of early T-cell progenitors (CD44-positive/CD25-negative/CD4 and CD8 double-negative). A study utilizing mitochondrial BH3 profiling found that ETP-ALL is BCL-2-dependent for its anti-apoptotic activity. This provided a rationale for treatment of ETP-ALL with the specific BCL-2 inhibitor, venetoclax. The sensitivity of venetoclax towards ETP-ALL was demonstrated in patient-derived xenograft models of ETP-ALL leukemic cells (74, 75).

A case report showed promising therapeutic effects of combined bortezomib and venetoclax treatment in patients with relapse/refractory ETP-ALL, with the ability to achieve hematological response and bridge to allogenic HSC transplant (76). Another case report showed successful treatment of relapse ETP-ALL by combining the venetoclax and HAG regime (homoharringtonine, cytarabine, and G-CSF) and the patient remained in remission after allogenic HSC transplant (77).

Combination of venetoclax and conventional chemotherapy was able to achieve remission with MRD negativity and bridge to allogenic HSC transplant in adult patients with ETP-ALL as documented in a case report (78). A further case report demonstrated that combining venetoclax and decitabine could induce morphological remission and MRD negativity in primary refractory ETP-ALL in adult patients with *TP53* mutation (79).

A study recruited a total of 13 patients aged 20–75 years old with relapse/refractory T-ALL, including 5 patients with ETP-ALL. All patients were treated with venetoclax combined with chemotherapy or decitabine. Morphological remission was achieved in 60% of patients, while one patient had MRD negativity and that patient had ETP-ALL phenotype (80).

A phase 1 clinical study (NCT03181126) enrolled patients aged older than 4 years with relapse/refractory ALL, including 16 patients with T-ALL and treated patients with the combination venetoclax and navitoclax (a BCL-2/BCL-XL/BCL-W inhibitor). The overall response rate, including complete remission and complete remission with incomplete count recovery, was 38%. Among those patients with response, 66.7% achieved MRD negativity (81).

Additional phase I/II clinical trials are being conducted evaluating treatment of relapse/refractory T-ALL in adult patients using venetoclax combined with chemotherapy or other novel agents (NCT03808610, NCT03504644, NCT03808610, and NCT04752163). A safety and pharmacokinetics study of venetoclax is ongoing for relapse/refractory T-ALL in pediatric and young adults (NCT03236857). Venetoclax appears to be a promising agent against ETP-ALL either as monotherapy or combined with other agents. Data from various clinical studies will be available in the near future to delineate the role of venetoclax in treatment of ETP-ALL.

### Ruxolitinib

*IL7R* mutations lead to hyperactivation of the IL7 pathway and cause resistance to steroid treatment. Hyperactivation of the JAK/STAT pathway is also observed in response to IL7 pathway stimulation (55). Upregulation of JAK/STAT pathway proteins and activities is also observed in ETP-ALL patients with *PRC2* mutations, which is prevalent in patients with ETP-ALL. Moreover, JAK/STAT pathway hyperactivation is a common finding in patient samples of ETP-ALL (82). Therefore, ruxolitinib, an inhibitor for both JAK1 and JAK2, is a promising agent for ETP-ALL treatment (28, 57, 58). The administration of ruxolitinib was shown to reverse the effects of steroid resistance under the stimulation of the IL7 pathway (55). Ruxolitinib also significantly reduced leukemic burden in a patient-derived xenograft model of ETP-ALL (82). A randomized controlled trial (RCT) is currently recruiting patients with newly diagnosed pediatric ALL, including T-ALL, and they will be treated with conventional combined chemotherapy and ruxolitinib for those patients with JAK/STAT pathway aberration. The trial will investigate whether there will be improvement of EFS and OS after combination treatment with ruxolitinib (NCT03117751). More clinical studies are required to evaluate the role of ruxolitinib as monotherapy or combination with other agents in the treatment of ETP-ALL.

### Anti-CD33

CD33 is commonly expressed in AML, and the addition of the monoclonal antibody against CD33, gemtuzumab, into standard chemotherapy could improve survival in patients with AML without poor-risk cytogenetics (83). An earlier report showed that gemtuzumab was active against CD33-positive B lymphoblastic leukemia, in both *in vitro* and *in vivo* models (84). ETP-ALL commonly present CD33 expression, which accounted for 67% of ETP-ALL in one study. Targeted therapy of anti-CD33 demonstrated cytotoxicity in an ETP-ALL cell line (14). However, larger-scale clinical trials to study the role of gemtuzumab in ETP-ALL are lacking.

### Anti-CD38

A study including 196 patients with T-ALL aged from 1 to 53 years, which consisted of 188 diagnostic samples and 35 relapsed samples, showed that CD38 was positive (using ≥20% positivity as the cutoff) in 97.9% and 82.9% of diagnostic and relapsed samples, respectively. The percentage of blasts positive for CD38 were similar in the ETP-ALL versus non-ETP-ALL subgroup. The results also showed that CD38 expression was stable even in relapsed samples (85). Another study including 10 patients with ETP-ALL showed that CD38 expression was stable over multiple courses of chemotherapy. Thus, targeting CD38 expression is an attractive approach for treatment of ETP-ALL. Treatment of CD38-expressing T-ALL with daratumumab was successful in the xenograft model, including seven patient-derived xenografts of ETP-ALL (86). Furthermore, case reports showed that daratumumab was effective in achieving morphological remission and MRD negativity after MRD relapse post-allogenic HSC transplant and extramedullary relapse in adult patients with blasts expressing CD38 (87–89). Another case report demonstrated that sequential administration of nelarabine and daratumumab was able to induce morphological remission and MRD negativity in patients after refractory to conventional chemotherapeutic induction regimens (90).

A phase 2 clinical trial recruiting 32 patients with relapsed/refractory CD38-positive T-ALL or B-ALL is ongoing. The trial plans to include patients aged 1–30 years and to be treated with daratumumab combined with chemotherapy (NCT03384654). Another phase 2 trial plans to include 96 patients aged 28 days to 18 years with relapsed AML or ALL (both B-ALL and T-ALL) and is currently ongoing, and patients will be treated with isatuximab, another monoclonal antibody against CD38 combined with chemotherapy (NCT03860844).

### Anti-CD123

CD123 is expressed in several hematological malignancies, including hairy cell leukemia (91), blastic plasmacytoid dendritic cell neoplasm (92), and AML (93). A study involving 30 patients with T-ALL found that CD123 was expressed in 43.3% of T-ALL cases, which was less frequent than in B-ALL. However, CD123 expression was more prevalent in ETP-ALL and early non-ETP-ALL phenotype, which constitute approximately 92.3% of T-ALL overall. Thus, CD123 is another novel target for therapy. A monoclonal antibody against CD123 was proven to be effective against B-ALL in a pre-clinical study (94). A phase I clinical trial will recruit 40 patients aged 12 years or above with CD123-positive hematological malignancies, including T-ALL to be treated with a single agent, flotetuzumab, a monoclonal antibody against CD123 (NCT04681105). Another phase I/II study (NCT03386513) will recruit 252 patients aged 18 years or above with the diagnosis of CD123-positive hematological malignancies including T-ALL to be treated with a single agent of monoclonal antibody against CD123 (IMGGN632).

### CAR-T therapy

Chimeric antigen receptor engineered T-cell (CAR-T) therapy is a promising therapy for treating B lymphoblastic leukemia (95, 96). However, since normal T cells and malignant T cells share a similar antigen profile, the utility of CAR-T therapy in treating T-cell hematological malignancies is limited. A study group using CRISPR/Cas9 technology has deleted CD7 and the T-cell receptor and engineered to target CD7 in malignant cells. A pre-clinical model showed excellent activity against T-ALL cell line xenograft model and a patient-derived xenograft model. A fratricide assay showed that minimal fratricide was detected (97, 98). These findings were very promising for CAR-T therapy in T-cell malignancies. Another pre-clinical study using CAR-T cells targets CD7 expression in leukemia cells by downregulating CD7 expression in CAR-T cells. The approach showed effective killing of primary ETP-ALL leukemic cells in patient-derived xenografts without significant fratricide (99). CAR-T cells targeting CD5 were also effective in killing primary T-ALL in a pre-clinical model (100).

Various phase I clinical trials investigating CD7-targeting CAR-T cells are recruiting patients with T-cell hematological malignancies including T-ALL and are ongoing (NCT03690011, NCT04004637, NCT04860817, and NCT04620655). A clinical trial of CD5 targeting CAR-T cells for T-cell malignancies, including T-ALL, is also ongoing (NCT03081910).

### Other Potential Novel Therapies

Drugs targeting epigenetic pathways, such as with hypomethylating agents azacytidine and decitabine, could be useful, because of the association of DNA hypermethylation with *PRC2* mutation (58). As suggested previously, despite the lower rate of *PRC2* mutations in adults, there is a higher rate of *DNMT3A* mutation that also supports the use of hypomethylating agents in adult patients (101). However, the use of hypomethylating agents in treating ETP-ALL had not been fully investigated in a pre-clinical model and no clinical studies are available that address this issue. Since ETP-ALL commonly presents mutations of genes involved in epigenetic regulation and *BCL2* overexpression, the combination of azacytidine or decitabine with venetoclax is a reasonable option as a novel combination strategy. Moreover, a study showed that azacytidine induced expression of the proapoptotic protein NOXA, which enhanced the susceptibility of leukemic cells to venetoclax-induced apoptosis (102). This combination therapy is a promising approach for treating AML (103). A case report described how combined decitabine and venetoclax was successful in treating a patient with refractory T lymphoblastic lymphoma, although the patient was diagnosed with the cortical T subtype of T lymphoblastic lymphoma (104). Another case series included six patients with relapse/refractory ETP-ALL treated with decitabine combined with G-CSF, low dose cytarabine, and aclarubicin. The treatment successfully brought the disease into control and allowed to bridge into allogenic HSC transplantation (105). Further pre-clinical and clinical studies to evaluate the combination of hypomethylating agents and venetoclax are needed.

Since both *FLT3*-TKD and *FLT3*-ITD mutations are common in ETP-ALL and the prevalence of *FLT3* mutations can be up to 35%, the use of an *FLT3* inhibitor also provides a novel treatment approach. However, there are limited pre-clinical studies to evaluate this hypothesis and there are no clinical studies to investigate the use of *FLT3* inhibitors in ETP-ALL (106). Sorafenib is a tyrosine kinase inhibitor employed to treat patients with *FLT3*-mutated AML with promising results (107). A study using an *FLT3*-transfected cell model to create an *FLT3*-mutated or *FLT3* wild-type phenotype in T-ALL cell lines showed sensitivity towards sorafenib in *FLT3*-mutated cell lines, although the cell lines used in that study did not exhibit the ETP-ALL phenotype (108).

Another promising novel therapy involves BET inhibitors. In a mouse model of loss-of-function mutations of *PRC2* resulting in RAS pathway hyperactivation, tumor growth of peripheral nerve sheath tumors was observed when coupled with another mutation, for example, of *NF1*. The tumor was sensitive to a BET protein inhibitor in that study (109). Recent pre-clinical studies on mouse models of ETP-ALL showed the efficacy of BET inhibitors for treating ETP-ALL associated with *PRC2* mutation (48). Again, this approach has not been evaluated in clinical settings.

Given the growing knowledge of underlying genetics and molecular mechanisms of ETP-ALL, various potential novel therapies are available and some have demonstrated efficacy in treating ETP-ALL in pre-clinical models. Data from clinical studies are emerging. More comprehensive pre-clinical studies and large-scale clinical studies of novel therapies, either used as monotherapy or combined with other agents, are needed to establish their role in treating ETP-ALL.

## Conclusion

ETP-ALL is a distinct subtype of T-ALL with a unique immunophenotype and genetic profile. The clinical course is aggressive and management of patients with ETP-ALL is challenging. The outcome of patients is poorer than that of other subtypes of T-ALL in previous studies. The diagnosis of ETP-ALL is also challenging. Despite the limitations of flow immunophenotyping for diagnosis, our proposed definition, which is based on the WHO classification and knowledge of T-cell maturation, will help daily clinical practice for diagnosis. In the future, data from genetic profiling will give us greater insight into acute leukemia classification. Further research is needed to incorporate the data from genetic profiling for subclassification of different types of acute leukemia, including ETP-ALL, into clinical practice. A new classification system based on immunophenotype and genetic profiling may be helpful for disease prognostication and treatment planning (12).

The cellular origin of ETP-ALL is hypothesized to derive from early thymic progenitors. This hypothesis was also verified by pre-clinical studies. With the greater understanding of molecular pathogenesis of various genetic aberrations of ETP-ALL, the exact mechanisms of leukemogenesis will become clearer. However, further studies are needed, especially to dissect the molecular pathogenesis of DNA hypermethylation in leukemogenesis of ETP-ALL.

The clinical management of ETP-ALL is challenging, and the outcome of patients was poor in early patient cohorts. The practice of risk-adapted treatment and allogenic HSC transplantation improved outcomes of those patients in recent clinical studies. However, larger-scale clinical studies are required to establish the role of allogenic hemopoietic stem cell transplant in improving the outcome of patients with ETP-ALL.

Novel therapies are desperately needed by patients with relapse/refractory disease or those who cannot tolerate standard chemotherapeutic treatment. Based on the understanding of molecular pathogenesis, venetoclax and ruxolitinib have been revealed as effective treatments for ETP-ALL in pre-clinical models and some patients have been successfully treated by venetoclax or its combination with other agents. However, larger-scale clinical studies are needed to better evaluate these agents for treatment of ETP-ALL.

Immunotherapy including monoclonal antibodies and CAR-T therapy is emerging as a successful approach for treating relapse/refractory B-ALL. These therapies demonstrated efficacy in a pre-clinical model of ETP-ALL. With the advance of CRISPR/Cas9 technology, CD5- and CD7-targeted CAR-T cells were developed, and they are being introduced into clinical trials, together with monoclonal antibodies against CD33, CD38, and CD123.

Epigenetic aberrations and *FLT3* mutations are common in ETP-ALL, which rationalizes the use of hypomethylating agents, BET inhibitors, and *FLT3* inhibitors as reasonable novel therapies for ETP-ALL. However, these approaches have not been extensively studied, even in pre-clinical models. There is an urgent need for evaluating these agents in treating ETP-ALL, given the current understanding of molecular pathogenesis.

## Author Contributions

C-FS wrote the article and conceptualized the article. P-hM wrote the article. Both authors reviewed the article. All authors contributed to the article and approved the submitted version.

## Conflict of Interest

The authors declare that the research was conducted in the absence of any commercial or financial relationships that could be construed as a potential conflict of interest.

## Publisher’s Note

All claims expressed in this article are solely those of the authors and do not necessarily represent those of their affiliated organizations, or those of the publisher, the editors and the reviewers. Any product that may be evaluated in this article, or claim that may be made by its manufacturer, is not guaranteed or endorsed by the publisher.
